# DICE: Dual mTorc Inhibition in advanCed/recurrent Epithelial ovarian cancer resistant to standard treatment—a study protocol for a randomised trial investigating a novel therapy called TAK228

**DOI:** 10.1186/s13063-022-06201-3

**Published:** 2022-04-05

**Authors:** Francesca Fiorentino, Jonathan Krell, Consuelo Nohpal de la Rosa, Lee Webber

**Affiliations:** 1 King’s College London, London, UK; 2 Imperial College London, London, UK

**Keywords:** Ovarian cancer, Platinum-resistant, TAK228, Weekly paclitaxel

## Abstract

**Background:**

The standard initial treatment for ovarian cancer is surgery and platinum-based chemotherapy and potentially maintenance therapy with avastin or inhibitors of poly-ADP ribose polymerase (PARP). While a proportion of women are cured by this approach, the vast majority will relapse and become resistant to platinum chemotherapy either initially or on subsequent treatment. There is an unmet need to improve response to treatment and quality of life in these women. TAK228 is a novel therapy that can be added to standard treatment in the participant population and the aim of the DICE trial is to assess its effectiveness. Laboratory and clinical research has shown that these ovarian cancers may respond to the molecular target of a drug such as TAK228, and there have been studies using it in other advanced solid tumours including endometrial cancer.

**Methods:**

One hundred twenty-four eligible women will be recruited from participating research sites in the United Kingdom (UK) and Germany. Randomised participants will receive either weekly paclitaxel alone (standard treatment, *n* = 62) or TAK228 plus weekly paclitaxel (*n* = 62) until the cancer significantly worsens; there are significant adverse events or any other protocol-defined stopping criteria. Participants will be monitored for response to treatment (using radiological imaging), adverse events and quality of life during both randomised treatment and subsequent follow-up.

**Discussion:**

The primary objective/endpoint of the study is to compare the two treatments in terms of progression-free survival, or the length of time that each participant is alive without the cancer significantly worsening according to defined assessment criteria. If the addition of TAK228 to weekly paclitaxel chemotherapy is shown to significantly improve this statistically, and adverse events and quality of life are not significantly worse than standard treatment, then TAK228 plus weekly paclitaxel could potentially be taken forward within the context of a larger phase III trial.

**Trial registration:**

ClinicalTrials.govNCT03648489. Registered on 27 August 2018.

## Administrative information

Note: the numbers in curly brackets in this protocol refer to SPIRIT checklist item numbers. The order of the items has been modified to group similar items (see http://www.equator-network.org/reporting-guidelines/spirit-2013-statement-defining-standard-protocol-items-for-clinical-trials/).

**Table:** 

Title {1}	DICE: a randomised trial investigating a novel therapy called TAK228 in ovarian cancer resistant to standard treatment
Trial registration {2a and 2b}.	NCT03648489 ClinicalTrials.gov Registered 27^th^ August 2018 https://clinicaltrials.gov/ct2/show/NCT03648489
Protocol version {3}	Version 7.0 dated 21st July 2020
Funding {4}	Industry funding and free provision of investigational medicinal product (TAK228) by Takeda Pharmaceuticals International Co and Calithera Biosciences, Inc.
Author details {5a}	Dr Francesca Fiorentino, primary affiliation – King’s College London, francesca.fiorentino@kcl.ac.uk, secondary affiliation – Imperial College London Dr Jonathan Krell – Chief Investigator, Imperial College London, j.krell@imperial.ac.uk Mrs Consuelo Nohpal de la Rosa, Imperial College London, c.nohpal-de-la-rosa@imperial.ac.uk Mr Lee Webber – Corresponding author, Imperial College London, lee.webber@imperial.ac.uk
Name and contact information for the trial sponsor {5b}	Mr Lee Webber, lee.webber@imperial.ac.uk Deputy Cancer Clinical Trials Operations Manager Imperial College London
Role of sponsor {5c}	Study sponsor has crucial role in study design; collection, management, analysis, and interpretation of data; writing of the report; and the decision to submit the report for publication. Study funder has advisory role in study design and collection of data and must provide consent for any publication of data as per the contractual agreement with the study sponsor.

## Introduction

### Background and rationale {6a}

Sixty-five thousand six hundred women were diagnosed with ovarian, fallopian tube or primary peritoneal cancer (ovarian cancer, hereafter) in Europe in 2012 [1], and there were 42,700 deaths [2]. In the UK alone, there were 7400 new cases and 4100 deaths in 2014 [3]. At least 75% of ovarian cancers are diagnosed when the cancer is advanced and has already spread to the abdomen/lymph glands (stage 3) or other organs, e.g. the liver (stage 4) [4]. Standard treatment is surgery to remove the womb, ovaries and tubes, and chemotherapy using the platinum-based drug carboplatin and a taxane called paclitaxel. The chemotherapy can be given before and/or after surgery. A small proportion of women are cured, however, the cancer grows or comes back in 80–90% of cases (recurrent/relapsed ovarian cancer) [5]. Relapses occurring during chemotherapy, or within 6 months of it finishing, are known as platinum-refractory or platinum-resistant ovarian cancer. Other women eventually become platinum-resistant following at least one retreatment with platinum chemotherapy. Platinum-refractory and resistant women (hereafter platinum-resistant) are the population under study in the DICE trial.

There is a lack of effective treatments in this population. Weekly paclitaxel chemotherapy is the standard treatment option, with a variety of clinical trial data to support this approach. The proportion of women whose cancer significantly shrinks (partial response) or disappears (complete response) following treatment is between 25 and 55% [6]. However, just as women can become platinum-resistant, resistance to paclitaxel is a real issue and there is an unmet need to improve the effectiveness of chemotherapy by combining it with additional treatment. So-called targeted therapies are increasingly common because they block the growth and spread of cancer by interfering with specific processes involved in cell division and survival.

TAK228 is an oral (by mouth) targeted therapy that blocks the PI3K/AKT/mTOR pathway by inhibiting the molecules mTORC1 and mTORC2. As such it is known as an mTOR, or dual TORC inhibitor. Research has shown that the PI3K/AKT/mTOR pathway plays an important role in the process by which cancer becomes resistant to chemotherapy, as well as cancer cell growth, survival and blood supply [7–10]. It is also a pathway known to be particularly active in ovarian cancer. Using an mTOR inhibitor is therefore a logical strategy to prevent these processes.

TAK228 has been tested as a single agent (monotherapy) in animals and also in early phase trials for humans with advanced solid and haematological (blood) cancers. A study of TAK228 in combination with weekly paclitaxel +/− trastuzumab, in advanced solid cancers, showed a potential for tolerability and response [11]. Another study in endometrial cancer showed improved progression-free survival (the length of time the recipient is alive and the cancer does not significantly worsen) for the combination of TAK228 and weekly paclitaxel; however, this improvement was not found to be statistically significant [12].

It is essential to further test TAK228 and weekly paclitaxel; this time in platinum-resistant ovarian cancer. It is insufficient to merely test whether TAK228 improves the effectiveness of paclitaxel; it is also important to test whether chemotherapy improves the effectiveness of TAK228. The overall aim of the study is to investigate whether the addition of TAK228 to weekly paclitaxel in women with ovarian cancer resistant to platinum-based chemotherapy improves a range of outcomes, with the primary outcome of progression-free survival, estimated as the length of time that each participant is alive without the cancer significantly worsening according to defined assessment criteria.

Potential benefits justifying the research include:

- Greater effectiveness of TAK228 plus weekly paclitaxel compared to existing treatments for the participant population, including weekly paclitaxel alone;
- Increased effectiveness of both TAK228 and paclitaxel when the two drugs are given together;
- By collecting cancer tissue and blood samples while running the study, there is a potential to link response to treatment with biological markers that can be used to predict which women are likely to benefit from TAK228 plus weekly paclitaxel in future.

## Objectives {7}

Primary:

To compare the efficacy of the combination of TAK228 plus weekly paclitaxel to weekly paclitaxel alone based on progression-free survival (PFS) in women with ovarian cancer resistant to standard platinum-based chemotherapy. PFS is assessed by the Response Evaluation Criteria in Solid Tumours (RECIST, version 1.1) [13] based on radiological imaging and is defined as the time from randomisation to first evidence of disease progression or death due to any cause.

Secondary:

To compare the following efficacy outcomes between TAK228 plus weekly paclitaxel and weekly paclitaxel alone:

- PFS at 24 weeks
- Overall response rate (ORR), defined as a complete response (CR) or partial response (PR) to treatment according to RECIST
- Duration of response (DoR), defined as the time from study entry to change in response from CR or PR or stable disease (SD) to progressive disease (PD) according to RECIST
- Time to progression (TTP), defined as the time from study entry to first evidence of disease progression or death due to any cause
- Clinical benefit rate (CBR) at 4 months, defined as CR, PR or SD for > 4 months
- Response according to Gynecologic Cancer Intergroup (GCIG) Cancer Antigen 125 (CA125) criteria [14]
- Overall survival (OS), defined as time from study entry to death due to any cause or to study termination
- Safety and tolerability, as assessed by adverse events according to the Common Terminology Criteria for Adverse Events (CTCAE) version 4.03 [15]
- Quality of life as a participant reported outcome, assessed by the European Organisation for Research and Treatment of Cancer (EORTC) validated questionnaires QLQ-C30 and QLQ-OV28

Exploratory:

To collect, store and analyse blood samples, archival tumour samples and fresh tumour biopsies:

1. Biomarker characteristics including TP53, PTEN, PIK3CA, mTOR, KRAS, BRCA1/2, CCNE1, ER, PR, HER2 status in archival and fresh tumour tissue specimens and circulating tumour DNA (ctDNA);
2. Compare biomarker status/responses between TAK228 plus weekly paclitaxel and weekly paclitaxel alone and correlate these with response and survival

## Trial design {8}

**Fig. 1:**
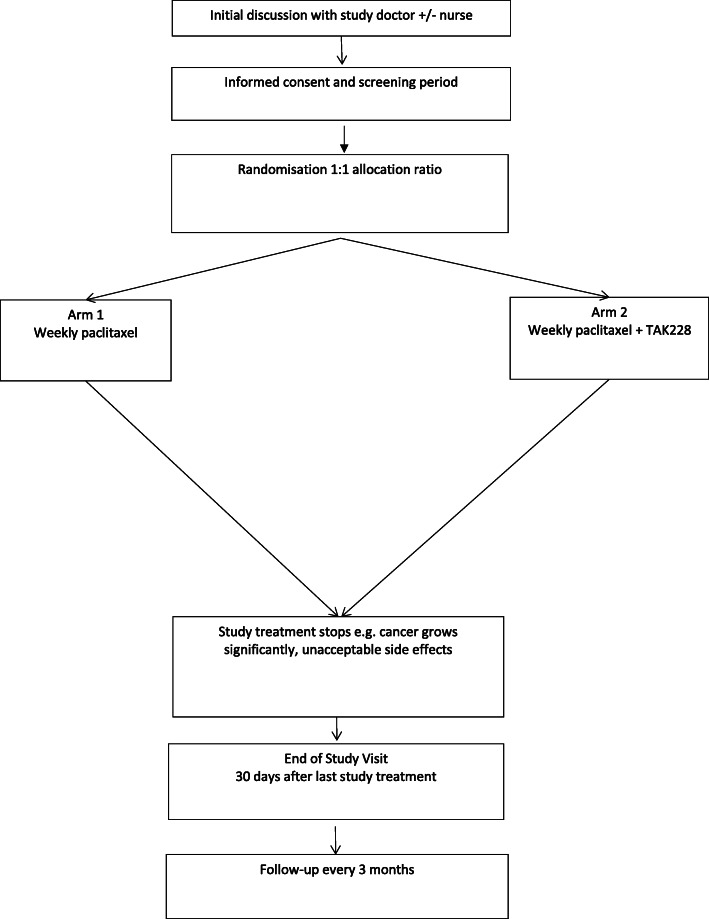
Trial scheme/flowchart

Phase II international multi-centre randomised parallel group, 1:1 allocation ratio, superiority framework, open label (Fig. 1).

## Methods: participants, interventions and outcomes

### Study setting {9}

Participating hospital research sites in the UK and Germany. A list of study sites can be obtained from the trial registration listing.

### Eligibility criteria {10}

Inclusion criteria:

1. Signed and dated written informed consent prior to admission to the study and initiation of any study procedures in accordance with The International Council for Harmonisation Good Clinical Practice guidelines and to the local legislation.
2. Females ≥18 years of age.
3. Pathological diagnosis of ovarian, fallopian tube or primary peritoneal cancer, of clear cell, endometrioid or high grade serous subtype or carcinosarcoma. Local tumour board/multi-disciplinary team histological review is required, and in mixed tumours, more than 50% endometrioid, clear cell or high grade serous elements are required to define the predominant histology.
4. Platinum-resistant (recurrence within 6 months of platinum treatment), or platinum refractory disease (recurrence during platinum treatment), patients having received at least one prior line of chemotherapy. Carboplatin and weekly paclitaxel are permitted as first-line therapy and/or as therapy for recurrent platinum-sensitive disease, i.e. prior to platinum-resistant relapse.
5. Measurable disease as per RECIST version 1.1 by computerised tomography (CT) or magnetic resonance imaging (MRI).
6. Fresh tumour biopsy during screening is compulsory, if judged technically feasible by radiologist, unless the local site is unable to collect the sample due to COVID-19 capacity restrictions.
7. Patients with a history of brain metastasis are eligible as long as all the following criteria are met: brain metastases must have been treated, have no evidence of progression or haemorrhage after treatment, have been off dexamethasone for 4 weeks prior to first study treatment and have no ongoing requirement for dexamethasone or anti-epileptic drugs.
8. Available blocks for immunohistochemistry and tissue microarray or, if no block is available, 20 ordinary unstained slides (5 μm sections) will be acceptable.
9. Eastern Cooperative Oncology Group (ECOG) performance status 0–2.
10. Adequate organ and bone marrow function.
11. Female patients who:

- Are postmenopausal for > 1 year before the screening visit OR
- Are surgically sterile OR
- If of childbearing potential, patient agrees to practise one of the following from informed consent to 90 days after the last dose of study treatment (or longer, as mandated by local labelling [e.g. Summary of Product Characteristics]):

i. Practise 1 highly effective method of contraception and 1 additional effective barrier method at the same time OR

ii. Practise true abstinence where this is in line with the preferred and usual lifestyle of the patient

12. For women of child-bearing potential, negative blood serum pregnancy test within 14 days prior to the first study treatment.
13. Able to swallow oral medication.

Exclusion criteria:

1. Previous treatment with PI3K, AKT, dual PI3K/mTOR inhibitors, mTORC1/2 inhibitors or mTORC1 inhibitors.
2. Prior weekly single agent paclitaxel.
3. Known allergy to paclitaxel and/or any excipients of investigational medicinal products that, in the investigator’s opinion, precludes study treatment on clinical and/or safety grounds.
4. Treatment with strong inhibitor/s and/or inducer/s of cytochrome P450 (CYP) 3A4 or CYP2C8 within 7 days of study treatment.
5. Central nervous system metastasis, for patients who have brain metastases, they will be eligible if their brain metastases must have been treated, have no evidence of progression or haemorrhage after treatment, have been off dexamethasone for 4 weeks prior to first study drug administration and have no ongoing requirement for dexamethasone or anti-epileptic drugs.
6. Other clinically significant co-morbidities, such as uncontrolled pulmonary disease, active central nervous system disease, active infection or any other condition that could compromise the patient’s participation in the study.
7. Known human immunodeficiency virus infection.
8. Known hepatitis B surface antigen-positive, or known or suspected active hepatitis C infection.
9. Any serious medical or psychiatric illness that could, in the investigator’s opinion, potentially interfere with the completion of treatment according to this protocol.
10. German sites only: Unable to be regularly followed up for any reason (geographic, familiar, social, psychological, housed in an institution, e.g. prison because of a court agreement or administrative order).
11. German sites only: Subjects that are dependent on the sponsor (and/or contracted body, e.g. contract research organisation) or investigational site as well as on the investigator.
12. Diagnosed or treated for another malignancy within 2 years before administration of the first dose of study treatment, or previously diagnosed with another malignancy and evidence of residual disease. Patients with nonmelanoma skin cancer or carcinoma in situ of any type are not excluded if they have undergone complete resection.
13. Breast feeding or pregnant.
14. Manifestations of malabsorption due to prior gastrointestinal surgery, gastrointestinal disease or for an unknown reason that may alter the absorption of TAK228. In addition, patients with enteric small bowel stomata are also excluded.
15. Treatment with any investigational products, chemotherapy or radiotherapy within 28 days, or major surgery within 21 days of study treatment.
16. History of any of the following within the last 6 months before administration of the first dose of study treatment:
  - Ischaemic myocardial event, including angina requiring therapy and artery revascularisation procedures
  - Ischaemic cerebrovascular event, including transient ischaemic attack and artery revascularisation procedures
  - Requirement for inotropic support (excluding digoxin) or serious (uncontrolled) cardiac arrhythmia (including atrial flutter/fibrillation, ventricular fibrillation or ventricular tachycardia)
  - Placement of a pacemaker for control of rhythm
  - New York Heart Association Class III or IV heart failure
  - Pulmonary embolism
17. Significant active cardiovascular or pulmonary disease.
18. Patients receiving systemic corticosteroids (either intravenous (IV) or oral steroids, excluding inhalers or low-dose hormone replacement therapy) within 1 week before administration of the first dose of study treatment.
19. Daily or chronic use of a proton pump inhibitor (PPI) and/or having taken a PPI within 7 days before receiving the first dose of study treatment.
20. Poorly controlled diabetes mellitus defined as glycosylated haemoglobin (HbA1c) > 7%; patients with a history of transient glucose intolerance due to corticosteroid administration may be enrolled in this study if all other inclusion/exclusion criteria are met.

### Who will take informed consent? {26a}

Informed consent will be obtained by appropriately trained and delegated investigator at an outpatient clinic appointment within the relevant participating hospital research site with the following assurances for each consented participant:

- Full and adequate oral and written information about the study including the background, purpose and risks/benefits of participation.
- Notification that they are free to withdraw from the study at any time.
- Opportunity to ask questions, allowed sufficient time to read and understand the information sheet and given at least 24 h to decide whether or not to take part
- Storage of the signed, dated informed consent form in the participant’s medical records and also the investigator site file.
- The participant receives a copy of the signed, dated informed consent form.

### Additional consent provisions for collection and use of participant data and biological specimens {26b}

The study-wide informed consent form is inclusive of all relevant use of participant for collection and use of participant data to include personal data; the mandatory collection of relevant biological specimens, e.g. archival tissue, tissue biopsy at screening if judged technically feasible and blood sample during screening, treatment and end of treatment.

### Interventions

#### Explanation for the choice of comparators {6b}

The comparator or control arm of this study is weekly paclitaxel, which is a standard treatment option in women with ovarian cancer that is resistant to platinum-based chemotherapy.

#### Intervention description {11a}

Arm 1: Weekly paclitaxel

Treatment according to 4 weeks/28 day cycles within administration at 80 mg/m^2^ full dose IV on days 1, 8 and 15 per cycle.

Arm 2: Weekly paclitaxel plus TAK228

Paclitaxel as per arm 1.

TAK228 administration at 4 mg full dose orally on days 2–4, 9–11, 16–18 and 23–25 per cycle.

#### Criteria for discontinuing or modifying allocated interventions {11b}

Protocol treatment discontinuation criteria are as follows:

- Confirmed disease progression and/or death
- Significant adverse events or unacceptable toxicities, including but not limited to significant adverse events or unacceptable toxicities that require discontinuation of study treatment according to the relevant dose modification algorithm
- Allergic reaction to study treatment
- Participant decision
- If the Investigator considers that a participant’s health will be compromised due to adverse events or concomitant illness that develop after entering the study
- Severe non-compliance to this protocol as judged by the Investigator
- Pregnancy and/or breast feeding/lactation

With respect to modification of randomised treatment by one or a combination of dose delay, omission or reduction, these are permitted for stipulated instances of the following adverse events:

- Neutropaenia
- Febrile neutropaenia
- Thrombocytopaenia
- Elevated serum creatinine
- Elevated liver function tests, i.e. bilirubin, alanine transaminase or aspartate transaminase
- Elevated clotting screen times, i.e. prothrombin and activated partial thromboplastin
- Hypersensitivity/allergic reaction
- Poorly controlled nausea, vomiting, diarrhoea, constipation
- Neuropathy
- Pneumonitis
- Hyperglycaemia
- Hyperlipidaemia
- Oral mucositis
- Rash
- Left ventricular dysfunction
- Corrected QT prolongation
- Other non-haematological toxicities (e.g. asthenia, weakness and fatigue)

#### Strategies to improve adherence to interventions {11c}

Weekly paclitaxel chemotherapy is given in an outpatient setting and is a standard treatment in the participant population, meaning that there are no significant anticipated challenges in participant adherence and/or compliance.

With respect to TAK228, participants randomised to receive this treatment are given detailed information and counselled at screening, including provision of a treatment diary that they are encouraged to complete. Participants are instructed to bring their completed diary and capsule bottles with them to all protocol scheduled visits so that they can be checked by the medical team responsible for their care, and any queries or problems can be discussed and raised with the central sponsor study team as needed.

All study deviations, whether relating to adherence and/or compliance to study treatment are recorded on the central electronic study database, with corrective and preventative actions recorded and followed up in order to mitigate any deemed risk/s to participant safety and/or data integrity.

#### Relevant concomitant care permitted or prohibited during the trial {11d}

Guidance regarding concomitant medications:

Prophylactic use of anti-emetic, anti-nausea, and anti-diarrhoeal medications is encouraged and may be used prior to first dose of study treatment (paclitaxel +/− TAK228), and as needed throughout the study prior to each dosing of study treatment and as clinically indicated per standard practice. When selecting an anti-emetic agent, drugs that do not have an effect on the QT interval, such as palonosetron, are preferred.

Histamine H_2_ receptor antagonists may be allowed if needed, provided that the histamine H_2_ receptor antagonist is not taken ≤ 12 h before and ≤ 6 h after TAK228 administration (Arm 2). Participants receiving histamine H_2_ receptor antagonists before study entry must not receive such medications ≤ 24 h prior to their first dose of TAK228. It is important that this adhered to in relation to paclitaxel treatment. Examples of histamine H_2_ receptor antagonists include ranitidine, famotidine and nizatidine. Cimetidine, a moderate CYP1A2 inhibitor, is not recommended as a first choice H_2_ receptor antagonist.

Neutralising antacid preparations (acid neutralisers) and calcium supplements are permitted except from 4 h before until 2 h after TAK228 administration (Arm 2). Some anti-gas preparations may also have antacid properties and should also not be permitted from 2 h before until 2 h after TAK228 administration.

Strong CYP1A2 inducers and/or inhibitors should only be administered with caution, at the discretion of the Investigator. Alternative treatment, if available, should be considered.

Paclitaxel should be administered with caution in participants receiving protease inhibitors as concomitant therapy. Post-chemotherapy supportive medications for paclitaxel, such as anti-emetics, can be given as per local policy providing these are not contraindicated.

Prohibited medications (Arms 1 and 2):

- Strong inhibitor/s and/or inducer/s of cytochrome P450 (CYP) 3A4 or CYP2C8

Prohibited medications (Arm 2):

- Other investigational medicinal product/s including PI3K, AKT, dual PI3K/mTOR inhibitors, mTORC1/2 inhibitors or mTORC1 inhibitors.
- Other anti-cancer therapies including chemotherapy, i.e. additional to paclitaxel given according to study protocol, immunotherapy, radioimmunotherapy, targeted agents, radiation or surgery.
- NB Palliative radiation for pain control of pre-existing lesions is allowed with sponsor approval. Bowel surgery and colostomy but not ileostomy are allowed.
- Systemic corticosteroids (either IV or oral steroids, excluding inhalers or premedication with dexamethasone prior to paclitaxel administration), unless necessary for treatment of TAK228-related adverse events, e.g. rash. Pre-medication for paclitaxel is required.
- Anti-epileptic drugs for participants with treated brain metastasis.
- Medications known to prolong the corrected QT interval, e.g. clarithromycin, antipsychotics, citalopram, amitriptyline and ondansetron.
- Concomitant administration of any protein pump inhibitor is not permitted during the study. Participants receiving such therapy before study entry must stop using the PPI > 7 days prior to their first dose of study treatment. Examples of PPIs include omeprazole, esomeprazole, pantoprazole, lansoprazole and rabeprazole.

No dietary restrictions will be imposed on study participants other than daily fasting for glucose monitoring. Participants who show evidence of hyperglycaemia during the study should be encouraged to follow a low carbohydrate diet.

#### Provisions for post-trial care {30}

Post-trial and/or ancillary care following completion of the randomly allocated treatment is at the discretion of the relevant investigator depending on the participant’s clinical and associated factors and corresponding standard of care. Compensation to those who suffer harm from trial participation is available through the study sponsor and/or the relevant participating hospital research site.

### Outcomes {12}

Primary:

PFS (median) assessed by RECIST, version 1.1, based on radiological imaging performed at baseline and then every 8 weeks during randomised treatment +/− at the end of treatment and then every 3 months during follow-up using CT or MRI scan. Defined as the time from randomisation to first evidence of disease progression or death due to any cause.

This efficacy outcome has clinical relevance as the aim of treating platinum-resistant ovarian cancer is disease control rather than cure since women will inevitably die of the cancer. Prolongation of PFS to a statistically significant degree within the weekly paclitaxel plus TAK228 arm when compared to weekly paclitaxel alone would make this approach worthy of investigation in a larger randomised clinical trial.

Secondary:

- PFS at 24 weeks (median) assessed by RECIST, version 1.1, based on radiological imaging performed at baseline and then every 8 weeks during randomised treatment until 24 weeks. Defined as the time from randomisation to first evidence of disease progression or death due to any cause;
- ORR (proportion) assessed by RECIST, version 1.1, based on radiological imaging performed at baseline and then every 8 weeks during randomised treatment +/− at the end of treatment and then every 3 months during follow-up using CT or MRI scan. Defined as a complete (CR) or partial response (PR) to treatment according to RECIST. The best overall response will be used in the analysis.
- DoR (median) assessed by RECIST, version 1.1, based on radiological imaging performed at baseline and then every 8 weeks during randomised treatment +/− at the end of treatment and then every 3 months during follow-up using CT or MRI scan. Defined as the time from study entry to change in response from CR or PR or stable disease (SD) to progressive disease (PD) according to RECIST.
- TTP (median) assessed by RECIST, version 1.1, based on radiological imaging performed at baseline and then every 8 weeks during randomised treatment +/− at the end of treatment and then every 3 months during follow-up using CT or MRI scan. Defined as the time from study entry to first evidence of disease progression or death due to any cause;
- CBR at 4 months (proportion) assessed by RECIST, version 1.1, based on radiological imaging performed at baseline and then every 8 weeks during randomised treatment +/− at the end of treatment and then every 3 months during follow-up using CT or MRI scan. Defined as CR, PR or SD for > 4 months.
- Response according to GCIG CA125 criteria (proportion) performed at baseline, during treatment, at the end of treatment and then every 3 months during follow-up. CA125 response according to GCIG criteria. A response according to CA125 has occurred if there is at least a 50% reduction in CA125 levels from a pre-treatment sample. The response must be confirmed and maintained for at least 28 days. Participants can be evaluated according to CA125 only if they have a pre-treatment sample that is at least twice the upper limit of normal and within 2 weeks prior to starting treatment.
- OS (median) assessed at baseline, throughout treatment and follow-up and defined as time from study entry to death due to any cause or to study termination.
- Safety and tolerability (proportion) assessed at baseline and throughout treatment and follow-up by adverse events according to the CTCAE version 4.03.
- Quality of life as a participant-reported outcome assessed at baseline, during and at the end of treatment by the EORTC validated questionnaires QLQ-C30 and QLQ-OV28.

### Participant timeline {13}

Participant timeline is shown in Tables 1, 2 and 3.

**Table 1:** Schedule of assessments for screening (all patients)

Time-point → Assessments ↓	Screening/baseline	
	Within 28 days	Within 14 days
Informed consent	X	
Inclusion/exclusion criteria	X	
Demographics	X	
Medical History	X	
German sites only: screening for HIV and hepatitis	X	
Vital signs		X
ECOG Performance Status		X
Complete physical examination		X
12-lead echocardiogram (ECG)	X	
Haematology		X
Biochemistry		X
Coagulation		X
Fasting serum glucose		X
Glycosylated haemoglobin (HbA1c)		X
Fasting lipid profile		X
Urinalysis		X
Creatinine clearance based on Cockcroft-Gault estimate, Wright Formula or urine collection		X
Blood serum pregnancy test		X
Research blood sample for genomic DNA		X
Archival tumour tissue	X	
Radiological imaging assessment (CT with contrast/MRI chest, abdomen and pelvis)	X	
CA125	X	
Research fresh tumour biopsy, if judged technically feasible by radiologist, unless the local site is unable to collect the sample due to COVID-19 capacity restrictions		X
Quality of Life Questionnaires (EORTC QLQ-C30 and EORTC QLQ-OV28)	X	
Adverse events (NCI CTCAE version 4.03)		X
Concomitant medications		X
Randomisation		X

**Table 2:** Schedule of assessments for Arm 1 patients

Time-point → Assessments ↓	Each treatment cycle			End of treatment visit	Follow-up
	Day 1	Day 8	Day 15	30 days post last treatment (±5 days)	Every 3 months (±5 days)
Vital signs	X	X	X	X	
ECOG Performance Status	X	X	X	X	
Physical examination	X	X	X	X	
Haematology	X	X	X	X	
Biochemistry	X	X	X	X	
Urinalysis	X		X Cycles 1 and 2 only	X	
Pregnancy test	X			X	
Research blood sample for ctDNA	X			X	
Radiological imaging assessment (CT with contrast/MRI chest, abdomen and pelvis)	Every 2 cycles/8 weeks (± 7 days)			X	X
CA125	X			X	X
Paclitaxel administration	X	X	X		
Quality of Life Questionnaires (EORTC QLQ-C30 and EORTC QLQ-OV28)	X			X	
Adverse events (NCI CTCAE version 4.03)	X	X	X	X	X
Concomitant medications	X	X	X	X	
Record of further treatment					X
Record of overall survival					X

**Table 3:** Schedule of assessments for Arm 2 patients

Time-point → Assessments ↓	Each treatment cycle				End of treatment visit	Follow-up
	Day 1	Day 2 (cycles 1 and 2 only)	Day 8	Day 15	30 days post last treatment (± 5 days)	Every 3 months (± 5 days)
Vital signs	X		X	X	X	
ECOG Performance Status	X		X	X	X	
Physical examination	X		X	X	X	
12-lead ECG	X				X	
Haematology	X		X	X	X	
Biochemistry	X		X	X	X	
Coagulation	X				X	
Fasting serum glucose	X				X	
Glycosylated haemoglobin (HbA1c)	X					
Fasting lipid profile	X				X	
Urinalysis	X			X Cycles 1 and 2 only	X	
Pregnancy test	X				X	
Research blood sample for ctDNA	X				X	
Radiological imaging assessment (CT with contrast/MRI chest, abdomen and pelvis)	Every 2 cycles/8 weeks (± 7 days)				X	X
CA125	X				X	X
In-home daily fasting glucose monitoring	X	X	X	X		
Paclitaxel administration	X		X	X		
TAK228 administration/compliance: administration at days 2–4, 9–11, 16–18 and 23–25 per cycle	X	X	X	X		
Quality of Life Questionnaires (EORTC QLQ-C30 and EORTC QLQ-OV28)	X				X	
Adverse events (NCI CTCAE version 4.03)	X	X	X	X	X	X
Concomitant medications	X	X	X	X	X	
Record of further treatment						X
Record of overall survival						X

### Sample size {14}

The sample size calculation is based on the hypothesis that the administration of TAK228 in combination with paclitaxel will increase median PFS by 2.15 months (i.e. from 4 to 6.15 months (hazard ratio of 0.65)) when compared to paclitaxel alone. The trial is designed to detect this difference over an 18-month accrual period (12-month recruitment and 6-month follow-up), with 80% power and a type I error rate of 10% (one-sided), no loss to follow-up and a minimum 6-month follow-up per participant.

Based on an initial sample size calculation using the SAS software and using the log-rank test, a total of 118 participants (59 per treatment arm) are needed to achieve 97 progression or death events. However, this does not consider the interim analysis for futility and safety. An interim analysis to assess safety and futility was planned when approximately 50% of the total progression events had occurred. Hence, the sample size was adjusted to account for the statistical analysis method used at the interim analysis stage [16, 17]. The group sequential method of O’Brien Fleming was used to select stopping rules for futility based on the primary endpoint analysis. Accordingly, in order to maintain power for the final analysis, allowing for one interim analysis for futility and safety, the number of events will need to be 102 (see published statistical analysis plan (SAP) for more details [https://trialsjournal.biomedcentral.com/articles/10.1186/s13063-021-05669-9]). To attain this number of events, the adjusted sample size is 124 participants (62 per treatment arm).

Patients who withdraw or are withdrawn before randomised treatment is administered will be replaced.

### Recruitment {15}

Timely recruitment in line with projected accrual rate will be achieved by:

- Thorough selection and feasibility assessment of prospective participating hospital research sites
- Close contact between central sponsor study team and participating hospital research sites to encourage discussion of progress and management of any perceived and/or actual recruitment barriers
- Use of study newsletters and site meetings
- Regular oversight of study progress including recruitment by trial management group and independent trial steering and data monitoring committees
- Close and regular contact between the central sponsor study team and the study funder

## Assignment of interventions: allocation

### Sequence generation {16a}

Randomisation will be on a 1:1 basis; it will be stratified and blocked, with random block sizes. There are three stratification factors: non-serous (clear cell or endometrioid) vs serous (high grade serous or carcinosarcoma) cancer, number of prior lines of chemotherapy (≤ 2 vs > 2 lines) and prior taxane interval (< 6 months vs ≥ 6 months or no prior taxane).

### Concealment mechanism {16b}

Randomisation of each participant is implemented centrally using the study electronic case report form database in Inform. There is no option available for manual randomisation. Even though the study is not blinded, it is not possible to have knowledge of allocation before patient enrolment in the study and in the Inform database. Allocation is kept concealed to the study team. Only the study statistician was unblinded at the time of the interim analysis for the corresponding closed IDMC report.

### Implementation {16c}

An independent statistician (external to the study team) will create the final randomisation list in accordance to the study protocol and the predefined sequence generation method (for stratification and blocks (see the section “Sequence generation {16a})). The randomisation list will be sent with password protection to the Inform team for embedding in the central system.

Baseline screening data for consented potential participants will be entered onto the central electronic study database and reviewed for eligibility by an appropriately trained and delegated member of the central sponsor study team. Eligible participants will be randomised within the database including randomised assignment of study treatment. Upon randomisation, each patient will be allocated a unique subject number to be used throughout the study.

## Assignment of interventions: blinding

### Who will be blinded {17a}

The study is open label with no blinding of participants, care providers, outcome assessors, data analysts, etc.

### Procedure for unblinding if needed {17b}

Not applicable, open label study.

## Data collection and management

### Plans for assessment and collection of outcomes {18a}

Collection and assessment of baseline and outcome data will occur according to the relevant outcome {12} and the participant timeline {13}.

Imaging data used for the assessment of relevant outcomes will be subject to independent external review to promote data quality.

The quality of life questionnaires used in the study are validated and thus reliable for the purposes of the relevant outcome analysis.

A copy of the data collection forms is held within the trial master file.

### Plans to promote participant retention and complete follow-up {18b}

There are no specific plans to promote participant retention and/or follow-up since treatment of ovarian cancer is based around outpatient visits to a secondary care hospital, and weekly paclitaxel, i.e. control arm is a standard treatment approach in these women. Furthermore, the follow-up frequency of the study, i.e. 3 monthly outpatient visits is reflective of standard clinical practice.

Participants who discontinue and/or deviate from the protocol, whether in terms of treatment or otherwise, will all be followed up for outcome data. It is particularly crucial that participants who discontinue randomised treatment without their cancer significantly worsening (progressive disease) continue to be followed up according to the protocol’s radiological imaging schedule, in order to accrue for both the primary as well as various response-based secondary endpoints.

### Data management {19}

Processes relating to data entry, coding, security and storage, as well as related processes to promote data quality, are documented in a separate data management plan and associated study-specific procedure manuals, e.g. electronic case report form completion guidelines and pharmacovigilance manual.

### Confidentiality {27}

Each participant will be identified by a unique subject number at randomisation. Month and year of birth will be collected along with ethnicity data. Individual patients will only ever be referred to by subject number and not identified in any trial-related correspondence, to include any published results.

All relevant data will be collected and maintained according to applicable data protection legislation and guidelines.

### Plans for collection, laboratory evaluation and storage of biological specimens for genetic or molecular analysis in this trial/future use {33}

The following biological specimens are collected from all participants as per the study-wide informed consent form taken prior to any study-specific procedures. Consent may be withdrawn by the participant at any time and any collected samples destroyed according to standard operating procedures:

- Baseline retrieval of archival tissue from ovarian cancer diagnosis/recurrence
- Baseline biopsy from ovarian cancer—one ambient and two frozen tumour cores per participant—if judged technically feasible by interventional radiologists, unless there are local radiology capacity issues due to COVID-19
- Baseline whole blood sample for analysis of genomic DNA
- Plasma samples at the start and end of treatment for analysis of ct DNA

In line with the aforementioned study-wide informed consent, these biological specimens will be stored at Imperial College London and examined using genetic and molecular analysis in order to assess the exploratory endpoints.

Analyses will be undertaken by an accredited central laboratory. Results of these analyses will not be included in the Clinical Study Report, but reported separately according to standard ethical and regulatory guidelines.

Any residual biological specimen material will then be transferred to the Imperial College Healthcare Tissue Bank and retained for future use in ethically approved research.

## Statistical methods

### Statistical methods for primary and secondary outcomes {20a}

The statistical analysis is detailed in the published Statistical Analysis Plan (SAP) [REFERENCE].

All analyses will be conducted on the intention to treat population unless otherwise specified. The statistical significance for the primary outcome is set at 11.005% (one-sided) to allow for an interim analysis of the primary endpoint (details in SAP [REFERENCE]). For the comparison between the two treatment arms (paclitaxel alone or paclitaxel plus TAK228) for all secondary endpoints, a two-tailed 5% significance level will be used.

Primary endpoint: The primary analysis of progression-free survival (PFS) will be a log-rank test adjusted by the stratification factors to estimate the difference in PFS between groups. Also, a Cox proportional hazards model adjusted by age and the stratification factors will be used to examine the effects of age and stratification factors.

Secondary endpoints: For the time to event secondary endpoints (PFS at 24 weeks, DoR, TTP and OS), the analysis will be performed using a two-sided stratified log-rank test for the comparison between treatment arms. Difference in ORR and CBR will be estimated using Cochran-Mantel Haenszel chi-square test.

All safety variables (AEs and SAEs) as well as vital signs, ECG, physical examination, laboratory parameters, adverse events and serious adverse events will be summarised (frequency and percentage), at patient level and AE level, by relationship to study treatment, severity, site, arm and country.

The score for both quality-of-life questionnaires, QLQ-C30 and QLQ-OV28, will be calculated using the European Organisation for Research and Treatment of Cancer (EORTC) scoring system. Mixed effect models of each dimension (15 for the QLC-30 and 7 for the QLQ-OV28, see SAP [REFERENCE]) of the quality of life (QoL) overall score will be used to investigate changes over time.

The final analysis will be performed once the data are cleaned, and the database is hard locked. All the statistical analyses will be performed using STATA/SE 17.

The detailed SAP has been published separately (REFERENCE).

### Interim analyses {21b}

An interim analysis was performed during the study, after 49 PFS events (approximately 50% of the expected events) occurred. Based on the results of the interim analysis, the IDMC advised to continue the study to completion.

A detailed interim analysis plan has been published within the SAP (REFERENCE).

### Methods for additional analyses (e.g. subgroup analyses) {20b}

No additional analyses are planned.

### Methods in analysis to handle protocol non-adherence and any statistical methods to handle missing data {20c}

#### Non-adherence

Sensitivity Analysis will be used to handle non-adherence to treatment protocol. This analysis will exclude patients with protocol violation/deviation/s due to non-adherence to randomised treatment.

#### Missing data

Reasons for missingness of the primary outcome data and the relationship to the treatment will be investigated. There will be no data imputation for missing data for the primary endpoint.

To determine the effect of COVID-19, the baseline characteristics at randomisation will be summarised for the population recruited before and after COVID-19 and any protocol deviations/violations related to COVID-19 will be monitored. Missing data are expected for some patients as a consequence of the COVID-19 pandemic.

### Plans to give access to the full protocol, participant-level data and statistical code {31c}

Not applicable, no current plans. Any future access will be subject to relevant standard operating procedures and contractual agreement between sponsor and funder.

## Oversight and monitoring

### Composition of the coordinating centre and trial steering committee {5d}

The TMG is composed of the Chief Investigator, Clinical Trial Coordinator, Clinical Trial Monitor, Operations Manager, Senior and Study Statistician, lead investigator in Germany and the principal investigator at the top recruiting UK site. The role and responsibility of the TMG is to enable day to day support of the trial, to include safety review of adverse events and protocol deviations/violations. The TMG will ideally meet at least 6 monthly.

The TSC is composed of an Independent Chair, Independent Clinician and Independent Patient Representative. The role and responsibility of the TSC is to provide independent support to the TMG, to include monitoring of recruitment, review of any significant substantial amendments, safety review of adverse events and protocol deviations/violations. The TSC is also executive to the IDMC and ultimately has the power to recommend closure of the study if there are significant concerns around its delivery in a safe, scientifically viable and timely manner. The TSC will ideally meet at least annually. Each member signs a Charter held in the trial master file, including declaration of any conflicting interests.

### Composition of the data monitoring committee, its role and reporting structure {21a}

The IDMC is composed of an Independent Chair, Independent Clinician and Independent Statistician. The role and responsibility of the IDMC is to independently review the safety and data quality aspects of the study, including interim analysis, and to provide recommendations to the TSC based on this independent review. The IDMC is independent from the sponsor and will ideally meet at least annually. Each member signs a Charter held in the trial master file, including declaration of any conflicting interests.

### Adverse event reporting and harms {22}

Collection of adverse event data will occur according to the relevant outcome {12} and the participant timeline {13}.

Adverse events will be assessed according to CTCAE version 4.03.

Adverse events will be reported via the study database, the electronic case report form. Serious adverse events will be reviewed by the Chief Investigator.

Specific anticipated adverse events should be managed according to relevant guidance within the study protocol.

### Frequency and plans for auditing trial conduct {23}

The study is subject to audit by the Imperial Clinical Trials Unit, specifically the Quality Assurance team. Two study specific audits have been conducted thus far, in addition to a wider laboratory audit that included this particular study. All findings have been managed with appropriate corrective and preventative actions, with no significant impact upon either participant safety or data integrity. While part of the sponsor these processes are independent of the TMG, the core study team and study investigators, and according to relevant standard operating procedures.

### Plans for communicating important protocol amendments to relevant parties (e.g. trial participants, ethical committees) {25}

Any important protocol amendments, e.g. changes to eligibility criteria, outcomes and analyses, will be subject to Research Ethics Committee, Health Research Authority and Regulatory Authority approvals and notified to investigators according to standard operating procedures. Such amendments may require updating of trial information held in public registries, etc.

Where the amendment is likely to have a significant impact on participants, this will be communicated to the participants themselves in the form of a letter that has been ethically approved. In exceptional circumstances, participants may need to be re-consented according to updated and ethically approved participant information.

Approved protocol amendments for the study at the time of submission are summarised as follows:

- Amendment to end of trial definition to ensure continued study treatment for participants receiving prolonged clinical benefit, as well as clarifying specific identifiable information to be collected in compliance with data protection legislation and guidelines;
- Amendment to add two eligibility criteria required for participating German research sites;
- Amendment to update the lead German investigator;
- Amendment to clarify eligibility criteria, prescribing/dispensing study treatment, management of dose interruptions, treatment breaks and in-home monitoring of fasting blood glucose (Arm 2 patients);
- Amendment to inclusion and exclusion criteria; additional information on potential risks; clarification of the circumstances under which the trial will be stopped; further clarification of in home glucometer monitoring (Arm 2 patients); clarification regarding pre, concomitant and restricted medications; addition to and clarification of the dose modification algorithms to manage specific adverse events; and clarification regarding discontinuation of study treatment due to unacceptable adverse events
- Amendment to waive the requirement for a screening tissue biopsy where there are capacity issues due to COVID-19
- Amendment to sample size and statistics relating to interim analysis and replacement of participants who are withdrawn from the study prior to receiving treatment, clarification on reportable SAEs and an update to the lead German investigator

## Dissemination plans {31a}

Trial results will be disseminated as follows:

- Publication in a peer review journal article, and associated oral presentations to the scientific community where relevant.
- Reporting on Clinicaltrials.gov and, where required, the European Medicines Agency database.
- Lay summaries on charity websites, e.g. Cancer Research UK and ovarian cancer-specific charities. The content of these lay summaries may be subject to advice from a public focus group.
- To trial participants in the form of a lay summary or similar, with potential input from a public focus group.

Dissemination must be according to the contractual agreement between sponsor and funder.

## Discussion

The study has been affected by the COVID-19 pandemic, with all UK hospital sites required to temporarily close. This required pragmatism from the study team in order to reopen sites in a timely manner and manage the subsequent lengthening of the recruitment period. Actions included a COVID-19 risk assessment, submission of a substantial amendment to waive the requirement for a screening tissue biopsy where there are capacity issues due to COVID-19 and submission and approval of a costed extension request from the study funder. While recruitment recommenced at a reduced and delayed rate, overall the study has recovered and is highly likely to deliver on its revised targets. Standard recruitment strategies have continued throughout this difficult period (see the section “Recruitment {15}”).

## Trial status

The current version of the protocol is 7.0 dated 21 July 2020. Recruitment began on 21 September 2018 and completed in July 2021 with a total of 134 participants. Follow-up is ongoing. It has not been possible to submit earlier due to the pressures created by the COVID-19 pandemic and a need to focus on keeping recruitment and general study operations running smoothly, as detailed in the “Discussion” section. We feel that publication of the protocol at this stage remains relevant to demonstrate the ability of studies to continue despite these unprecedented times. Moreover, the study will remain in active follow-up at least until the second quarter of 2022 and then enter a 6-month period of data cleaning, meaning that there is a significant period of time until the end of trial declaration and final analysis.

## Abbreviations

AE: Adverse event
AKT: Protein kinase B
BRCA: Breast cancer gene
CA125: Cancer antigen 125
CCNE1: Cyclin E1
CR: Complete response
CT: Computerised tomography
ctDNA: Circulating tumour DNA
CTCAE: Common Terminology Criteria for Adverse Events
CYP: Cytochrome P450
DoR: Duration of response
ECG: Electrocardiogram
ECOG: Eastern Cooperative Oncology Group
EORTC: European Organisation for Research and Treatment of Cancer
ER: Estrogen receptor
GCIG: Gynecologic Cancer InterGroup
H_2_: Hydrogen gas
HbA1c: Glycosolated haemoglobin
HER2: Human epidermal growth factor receptor 2
IDMC: Independent Data Monitoring Committee
IV: Intravenous
KRAS: Kirsten rat sarcoma viral oncogene homolog
MDT: Multidisciplinary team meeting
MRI: Magnetic resonance imaging
mTOR: Mechanistic target of rapamycin
mTORC: Mechanistic target of rapamycin complex
OS: Overall survival
PARP: Poly-ADP ribose polymerase
PD: Progressive disease
PFS: Progression-free survival
PI3K: Phosphoinositide 3-kinase
PIK3CA: Phosphatidylinositol-4,5-Bisphosphate 3-Kinase Catalytic Subunit Alpha
PPI: Protein pump inhibitor
PR: Partial response
PR: Progesterone receptor
PTEN: Phosphatase and tensin homolog
RECIST: Response Evaluation Criteria in Solid Tumours
SAE: Serious adverse event
SAP: Statistical Analysis Plan
SD: Stable disease
STATA/SE: Software for Statistics and Data Science/Standard Edition
TMG: Trial Management Group
TP53: Tumour protein 53
TSC: Trial Steering Committee
TTP: Time to progression
UK: United Kingdom
